# GlycA, a marker of protein glycosylation, is related to albuminuria and estimated glomerular filtration rate: the ELSA-Brasil study

**DOI:** 10.1186/s12882-017-0779-z

**Published:** 2017-12-20

**Authors:** Silvia M. Titan, Roberto Pecoits-Filho, Sandhi M. Barreto, Antônio Alberto Lopes, Isabela J. Bensenor, Paulo A. Lotufo

**Affiliations:** 1 Nephrology Unit, Department of Medicine, Medical School, University of Sao Paulo, Av Dr Enéas de Carvalho Aguiar 255, Cerqueira César, São Paulo - SP 05403-000 Brazil; 2 Center for Clinical and Epidemiologic Research, Hospital Universitario, University of São Paulo, Av Prof. Lineu Prestes 2565, Butantã, São Paulo - SP 05508-000 Brazil; 3 Department of Medicine, Medical School, Pontifícia Universidade Católica do Paraná, R. Imac. Conceição 1155. Prado Velho, Curitiba, PR 80215-901 Brazil; 4 Department of Social and Preventive Medicine, Medical School, Universidade Federal de Minas Gerais, Av. Prof. Alfredo Balena 190. Santa Efigênia, Belo Horizonte, MG 30130-100 Brazil; 5 Clinical Epidemiology and Evidence Based-Medicine, University Hospital Professor Edgard Santos, Federal University of Bahia, Rua Augusto Viana, sn°. Canela, Salvador, BA 40110-060 Brazil; 6 General Medicine Unit, Department of Medicine, Medical School, University of Sao Paulo, Av Dr Enéas de Carvalho Aguiar 255, Cerqueira César, São Paulo - SP 05403-000 Brazil

**Keywords:** GlycA, Protein glycosylation, CKD, Albuminuria

## Abstract

**Background:**

Systemic inflammation has been implicated in several chronic diseases. GlycA is a new nuclear mass resonance (NMR) spectroscopy-derived biomarker of systemic inflammation that reflects protein glycosylation. We evaluated the association of GlycA with albuminuria and eGFR in the ELSA-Brasil Study.

**Methods:**

The cross-sectional association between GlycA (automated NMR LipoProfile(®) test spectra, LabCorp, Raleigh, NC), and overnight 12 h–albuminuria and CKD-EPI eGFR was evaluated among 5050 participants.

**Results:**

GlycA was higher among older, women, smokers, alcohol abstemious, obese and in those with diabetes, hypertension or dyslipidemia. In addition, both eGFR and albuminuria were associated to GlycA. In linear regression, GlycA was independently associated with log albuminuria (B 0.03; 95%CI 0.02–0.04, *P* < 0.0001, per 1sd increase) and inversely related to eGFR (B -0.53; 95%CI -0.99 – -0.07, *P* < 0.02), even after adjustments including hsCRP. In logistic regression, GlycA was independently related to the risk of A2 or A3 albuminuria (OR 1.42, 95%CI 1.27–1.57, *p* < 0.0001, per 1sd increase), of having an eGFR < 60 ml/min/1.73m^2^ (OR 1.26, 95%CI 1.12–1.41, *p* = 0.0003, per 1 sd) or of a combined diagnosis of both conditions (OR 1.35, 95%CI 1.23–1.46, *p* < 0.0001, per 1 sd). In the ROC curve, GlycA had a higher AUC in comparison to hsCRP (AUC 0.67 vs. 0.62, *p* = 0.06) for the association with albuminuria A2 or A3.

**Conclusions:**

The present study demonstrates that GlycA is associated with albuminuria and eGFR, independently of major risk factors for CKD progression, including (and with a stronger association than) hsCRP. GlycA should be further evaluated in CKD progression.

## Background

GlycA is a NMR spectrometry-derived novel biomarker that reflects protein glycosylation. Although it potentially reflects N-acetylglucosamine residues on any circulating plasma protein, it has been shown that GlycA signal majorly reflects glycosylation of acute-phase proteins, particularly of α1-acid glycoprotein, haptoglobin, α1-antitrypsin, α1-antichymotrypsin, and transferrin [1]. Important changes both in the concentration of acute-phase reaction proteins as well as in their glycosylation pattern are known to occur in response to an inflammatory stimuli, being therefore important determinants of the GlycA signal. For that reason, GlycA is currently being studied as a new marker of systemic inflammation.

Recent studies have shown that GlycA is related to the risk of severe infection [2, 3], as well as to disease activity in lupus [4] and rheumathoid arthritis [5, 6]. In addition, GlycA has also a role in metabolic diseases and cardiovascular events and mortality. In prospective studies, GlycA has been related to overall, cardiovascular and cancer-related mortality [7–10]. It has also been related to the risk of incident diabetes [11], to insulin resistance, leptin/adiponectin [12] and to sodium excretion [13]. All the above mentioned associations of GlycA have been shown to be independent of C reactive protein (CRP), even though the use of the two biomarkers may provide additive prognostic information, at least for cardiovascular events [9].

Chronic kidney disease (CKD), as defined by reduced estimated glomerular filtration rate (eGFR) and/or the presence of albuminuria, is associated with low-grade inflammation, which was described as an important mechanism underlying CKD progression. However, studies evaluating the role of inflammatory biomarkers on CKD, including hsCRP, have yielded conflicting results [14–18]. Up to the present, GlycA has not been evaluated in the context of CKD. The aim of this study was to investigate the association of GlycA to albuminuria and eGFR in a Brazilian cohort of middle-aged men and women.

## Methods

The data used for the present investigation come from the Brazilian Longitudinal Study of Adult Health (ELSA-Brasil), a multicenter prospective cohort study designed primarily to identify risk factors and the natural history of diabetes and cardiovascular disease. The design and preliminary findings of this study have been published elsewhere [19, 20]. Briefly, 15,105 civil servants aged 35 to 74 years from six cities in Brazil were enrolled between August 2008 and December 2010 for baseline examination. Approvals from institutional review boards of all centers were granted, and all individuals signed informed consent. Since GlycA was measured only in the Sao Paulo sample, for the current analysis only data on the 5050 Sao Paulo participants was used.

Interviews and examinations were carried out by trained personnel with strict quality control [21]. Trained nurses measured patients’ weight, height, waist, and hip circumferences. Body mass index (BMI) was calculated by dividing the patients’ weight in kilograms by height in meters squared. Blood pressure was measured with the validated Omron HEM 705CPINT oscillometric device (Omron Co, Kyoto, Japan) after a 5-minute rest with the patient in a sitting position in a quiet, temperature-controlled room (20–24 °C). Three measurements were taken at 1-min intervals and the mean of these measurements was calculated. Baseline laboratorial measurements [22] were done after an overnight fast (urine and blood) and biological fluids were collected and frozen [23]. An oral glucose tolerance test (75 g) was performed in non-diabetic participants [22].

Diabetes was defined as previous medical history of diabetes, use of medication to treat diabetes, fasting plasma glucose ≥ 126 mg/dl, 2-h plasma glucose ≥200 mg/dl, or HbA1C ≥ 6.5% Glomerular filtration rate was calculated using the equations from the Chronic Kidney Disease Epidemiology Collaboration without correction for race [24]. Albuminuria was measured via nephelometry in a 12 h–overnight sample, and categories of albuminuria were defined according to KDIGO: albuminuria A1 as albumin-to-creatinine ratio < 30 μg/mg, albuminuria A2 as ≥ 30 μg/mg creatinine and albuminuria A3 as ≥ 300 mg/g creatinine. GlycA was measured by NMR spectrometry (*NMR LipoProfile*® test spectra, LabCorp, Raleigh, NC^1^) in plasma of the 5050 Sao Paulo participants.

In the descriptive analyses, Jonckheere-Terpstra test for ordered alternatives and chi-square test were used to test differences among quartiles of GlycA for continuous and categorical variables, respectively. Uni and multivariate linear regression models were built using albumin-to-creatinine ratio (log-transformed) and eGFR as the dependent variables. For the logistic regression models, the dependent variable was defined as albuminuria A2 or A3, eGFR < 60 ml/min/1.73m^2^ or both (albuminuria A2 or A3 and/or eGFR < 60 ml/min/1.73m^2^). Lastly, ROC curves were built on the diagnosis of albuminuria (albuminuria A2 or A3) and of eGFR < 60 ml/min/1.73m^2^ and the performances of hsCRP and GlycA were compared using c statistics (http://www.vassarstats.net/).

## Results

**Fig. 1:**
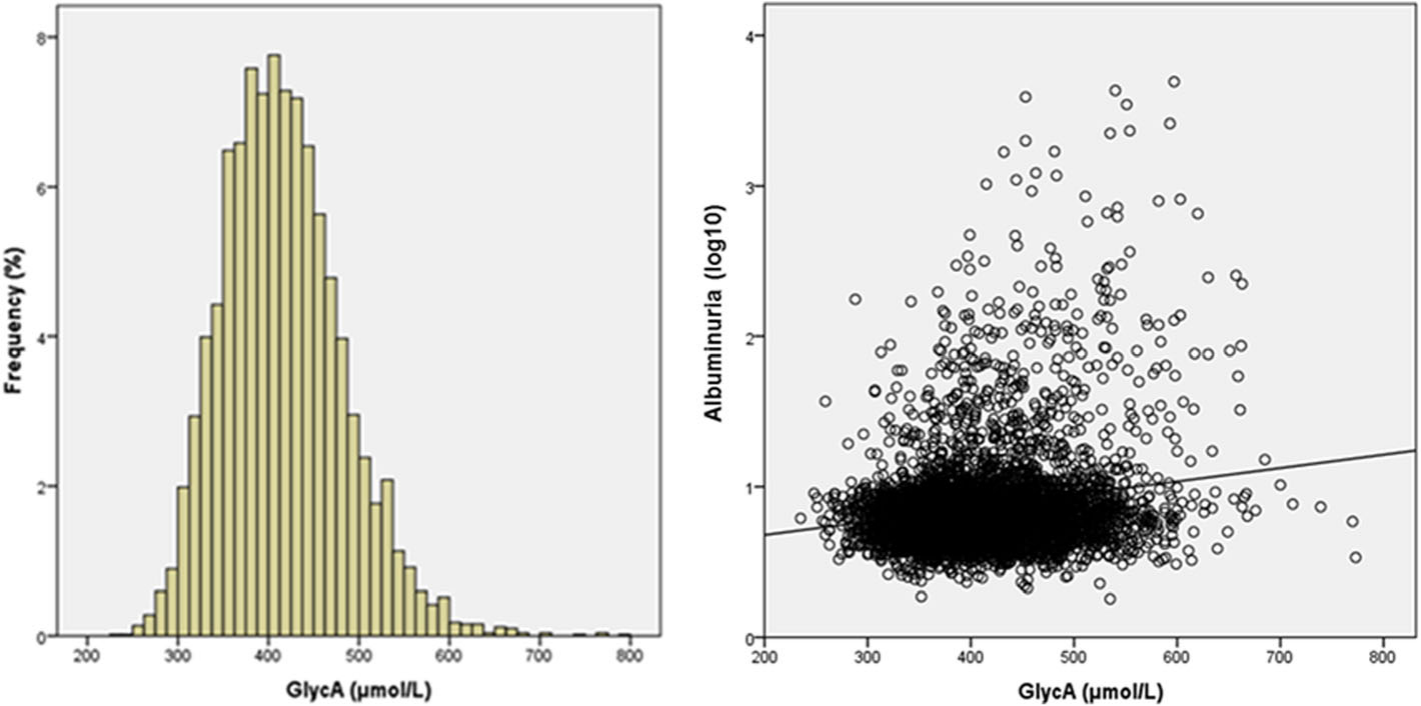
Histogram of GlycA and scatterplot between GlycA and albuminuria among 5050 participants

Among the 5050 participants, GlycA had a mean value of 416 ± 67 μmol/L (Fig. 1). In Table 1, descriptive clinical and laboratorial characteristics are shown according to the quartiles of GlycA. GlycA was positively related to several variables, including age, female sex, smoking, BMI, diabetes and insulin resistance biomarkers, blood pressure, albuminuria, hsCRP and lipids. It was also inversely related to alcohol consumption, eGFR and HDL-cholesterol. Figure 1 also shows the scatter plot between albuminuria and GlycA.

**Table 1:** Baseline clinical and laboratorial characteristics among 5050 participants according to quartiles of GlycA

	Quartile 1 GlycA ≤368 n=1262		Quartile 2 GlycA ≥369 - ≤410 n=1259		Quartile 3 GlycA ≥411 - ≤456 n=1266		Quartile 4 GlycA ≥457 n=1263		p**
Age (years; mean/std)	52	9	51	9	51	9	52	9	0.09
Sex (male; n / %)	702	0.56	602	0.48	548	0.43	472	0.37	< 0.001
Smoking status									< 0.001
Never (n/%)	752	0.60	672	0.53	665	0.53	574	0.45	
Former (n/%)	383	0.30	390	0.31	374	0.30	419	0.33	
Current (n/%)	127	0.10	197	0.16	227	0.18	270	0.21	
Alcohol									< 0.001
Never (n/%)	120	0.10	130	0.10	164	0.13	189	0.15	
Former (n/%)	242	0.19	224	0.18	268	0.21	288	0.23	
Current (n/%)	899	0.71	904	0.72	834	0.66	786	0.62	
Diabetes mellitus (n/%)	173	0.14	215	0.17	263	0.21	401	0.32	< 0.001
Self-reported myocardial infarction (n/%)	20	0.02	21	0.02	17	0.01	38	0.03	0.01
Self-reported CVD (n/%)	73	0.06	75	0.06	59	0.05	108	0.09	0.001
Self-reported stroke (n/%)	15	0.01	14	0.01	8	0.01	25	0.02	0.02
Body-mass index (kg/m^2^; mean/std)	25.5	4.3	26.9	4.3	27.9	4.6	29.3	5.5	< 0.001
Waist-to-hip ratio (mean/std)	.87	.09	.89	.09	.90	.08	.91	.09	< 0.001
Systolic blood pressure (mmHg; mean/std)	116.4	15.1	119.2	16.8	120.4	16.0	123.3	17.7	< 0.001
Diastolic blood pressure (mmHg; mean/std)	72.4	10.2	74.6	10.9	75.9	10.2	77.9	11.1	< 0.001
Leptin (ng/mL)	9.64	5.4–16.6	12.23	6.2–21.4	14.43	7.0–26.8	15.19	8.7–28.2	< 0.001
Hemoglobin (g/dL; mean/std)	14.5	1.4	14.4	1.4	14.3	1.3	14.1	1.4	< 0.001
Hematocrit (%; mean/std)	43.2	3.9	43.0	4.0	42.8	3.7	42.2	4.0	< 0.001
Glucose (mg/dL; mean/std)	106	18	109	25	112	28	120	43	< 0.001
Glucose among non-diabetics (mg/dL; mean/std)	102	8	103	9	104	8	105	8	< 0.001
HbA1C (%; mean/std)	5.2	0.7	5.4	0.9	5.5	0.9	5.8	1.3	< 0.001
Postload glucose 2 h (among non-diabetic, mg/dL; mean/std)	124	36	129	39	137	42	145	51	< 0.001
HOMA index* (median/IQR)	1.3	0.65–2.23	1.6	0.90–2.64	2.0	1.14–3.24	2.4	1.42–3.87	< 0.001
HOMA index among non-diabetics (median/IQR)	1.13	0.61–1.89	1.46	0.83–243	1.69	1.06–2.81	2.04	1.27–3.10	< 0.001
Total cholesterol (mg/dL; mean/std)	205	40	209	39	216	41	221	45	< 0.001
Triglycerides (mg/dL; median/IQR)	108	73	123	63	142	85	179	146	< 0.001
HDL-cholesterol (mg/dL; mean/std)	59	15	57	14	55	13	53	13	< 0.001
LDL-cholesterol (mg/dL; mean/std)	124	32	128	33	133	35	134	36	< 0.001
Uric acid (mg/dL; mean/std)	5.4	1.4	5.6	1.5	5.7	1.5	5.9	1.6	< 0.001
Aspartate Transaminase (AST) (U/L; mean/std)	25	12	26	12	26	17	25	10	< 0.001
Alanine Transaminase (ALT) (U/L; mean/std)	27	18	29	19	29	26	28	15	0.01
Gamma-Glutamyl Transferase (U/L, mean/std)	31	34	35	39	40	54	48	71	< 0.001
Liver right lobe (post_ant diameter) (cm; mean/std)	103	11	105	12	105	11	106	12	< 0.001
Potassio - Sangue (mEq/L)	4.5	0.3	4.6	0.4	4.6	0.3	4.6	0.4	< 0.001
Albumine-to-Creatinine ratio (μg/mg; median/IQR)	6	5–8	7	5–8	7	5–8	7	5–9	< 0.001
Estimated GFR CKD-EPI (ml/min/1.73 m^2^; mean/std)	83.1	14.3	82.9	15.2	83.0	15.3	81,.5	17.4	0.12
hs-C Reactive Protein (mg/L; mean/std)	1.0	1.2	1.8	1.9	2.8	3.0	6.0	7.3	< 0.001

^*^excluding insulin-users

^**^Jonckheere-Terpstra or chi-square tests

In Table 2, the linear regression models for albuminuria and eGFR as the dependent variables are shown. In the univariable model (model 1), GlycA shows a significantly positive relation to albuminuria and an inverse association to eGFR. In the subsequent models, the significant relation remains even after adjustments for potential confounding variables. In the last model (model 5), it is shown that the association of GlycA to albuminuria is independent of eGFR and vice-versa.

**Table 2:** Unadjusted and adjusted linear regression models on albuminuria and eGFR among 5050 participants of ELSA-Brasil

	B^*^	95%CI B^*^		*p* value
Albuminuria (log)				
Model 1: univariable				
GlycA	.05	.04	.06	< 0.0001
Model 2: sex and age adjusted				
GlycA	.04	.03	.05	< 0.0001
Model 3: sex, age, HbAlc, SBP and BMI adjusted				
GlycA	.03	.02	.04	< 0.0001
Model 4: sex, age, HbA1c, SBP, BMI, smoking, alcohol, LDL, HDL and hsCRP				
GlycA	.03	.02	.04	< 0.0001
Model 5: same model 4 + CKDEPI eGFR				
GlycA	.03	.02	.04	< 0.0001
CKD-EPI eGFR				
Model 1: univariable				
GlycA	-.79	-1.22	-.37	.0003
Model 2: sex and age adjusted				
GlycA	-.81	-1.19	-.43	< 0.0001
Model 3: sex, age, HbA1c, SBP and BMI adjusted				
GlycA	-.36	-.75	.04	.08
Model 4: sex, age, HbA1c, SBP, BMI, smoking, alcohol, LDL, HDL and hsCRP				
GlycA	-.60	-1.05	-.15	.01
Model 5: same model 4 + albuminuria				
GlycA	-.53	-.99	-.07	.02

^*^per 1sd increase of GlycA

In Table 3, uni and multivariate models on the diagnosis of albuminuria (albuminuria A2 or A3 versus A1), low eGFR (< 60 ml/min/1.73m^2^ versus > 60 ml/min/1.73m^2^) and both (albuminuria A2 or A3 and/or eGFR < 60 ml/min/1.73m^2^) are shown. In these models, GlycA remains significantly and independently related to albuminuria and/or eGFR.

**Table 3:** Unadjusted and adjusted logistic regression models on the diagnosis of albuminuria A2 or A3 (versus albuminuria A1), on eGFR < 60 ml/min/1.73m^2^ (versus eGFR > 60 ml/min/1.73m^2^) and on both (albuminuria or eGFR < 60 ml/min/1.73m^2^) among 5050 participants

	OR	95%CI OR		*p* value
Albuminuria A2 or A3 *n* = 248 (versus albuminuria A1 *n* = 4590) Per 1 sd increase				
Model 1: univariable				
GlycA	1.60	1.49	1.72	< 0.0001
Model 2: sex and age adjusted				
GlycA	1.64	1.52	1.76	< 0.0001
Model 3: sex, age, HbAlc, SBP and BMI adjusted				
GlycA	1.43	1.30	1.56	< 0.0001
Model 4: sex, age, HbA1c, SBP, BMI, smoking, alcohol, LDL, HDL and hsCRP				
GlycA	1.46	1.31	1.61	< 0.0001
Model 5: same model 4 + CKDEPI eGFR				
GlycA	1.42	1.27	1.57	< 0.0001
CKD EPI eGFR < 60 ml/min/1.73 m ^2^ *n* = 321 (versus > 60 ml/min/1.73 m2 *n* = 4519) Per 1 sd increase				
Model 1: univariable				
GlycA	1.30	1.19	1.41	< 0.0001
Model 2: sex and age adjusted				
GlycA	1.33	1.22	1.45	< 0.0001
Model 3: sex, age, HbA1c, SBP and BMI adjusted				
GlycA	1.24	1.12	1.36	< 0.0001
Model 4: sex, age, HbA1c, SBP, BMI, smoking, alcohol, LDL, HDL and hsCRP				
GlycA	1.32	1.18	1.46	< 0.0001
Model 5: same model 4 + albuminuria				
GlycA	1.26	1.12	1.41	.0003
Albuminuria and/or CKD-EPI eGFR < 60 ml/min/1.73 m ^2^ (*n* = 518 vs. 4433) Per 1 sd increase				
Model 1: univariable				
GlycA	1.38	1.36	1.45	< 0.0001
Model 2: sex and age adjusted				
GlycA	1.45	1.36	1.54	< 0.0001
Model 3: sex, age, HbA1c, SBP and BMI adjusted				
GlycA	1.28	1.19	1.38	< 0.0001
Model 4: sex, age, HbA1c, SBP, BMI, smoking, alcohol, LDL, HDL and hsCRP				
GlycA	1.35	1.23	1.46	< 0.0001

**Fig. 2:**
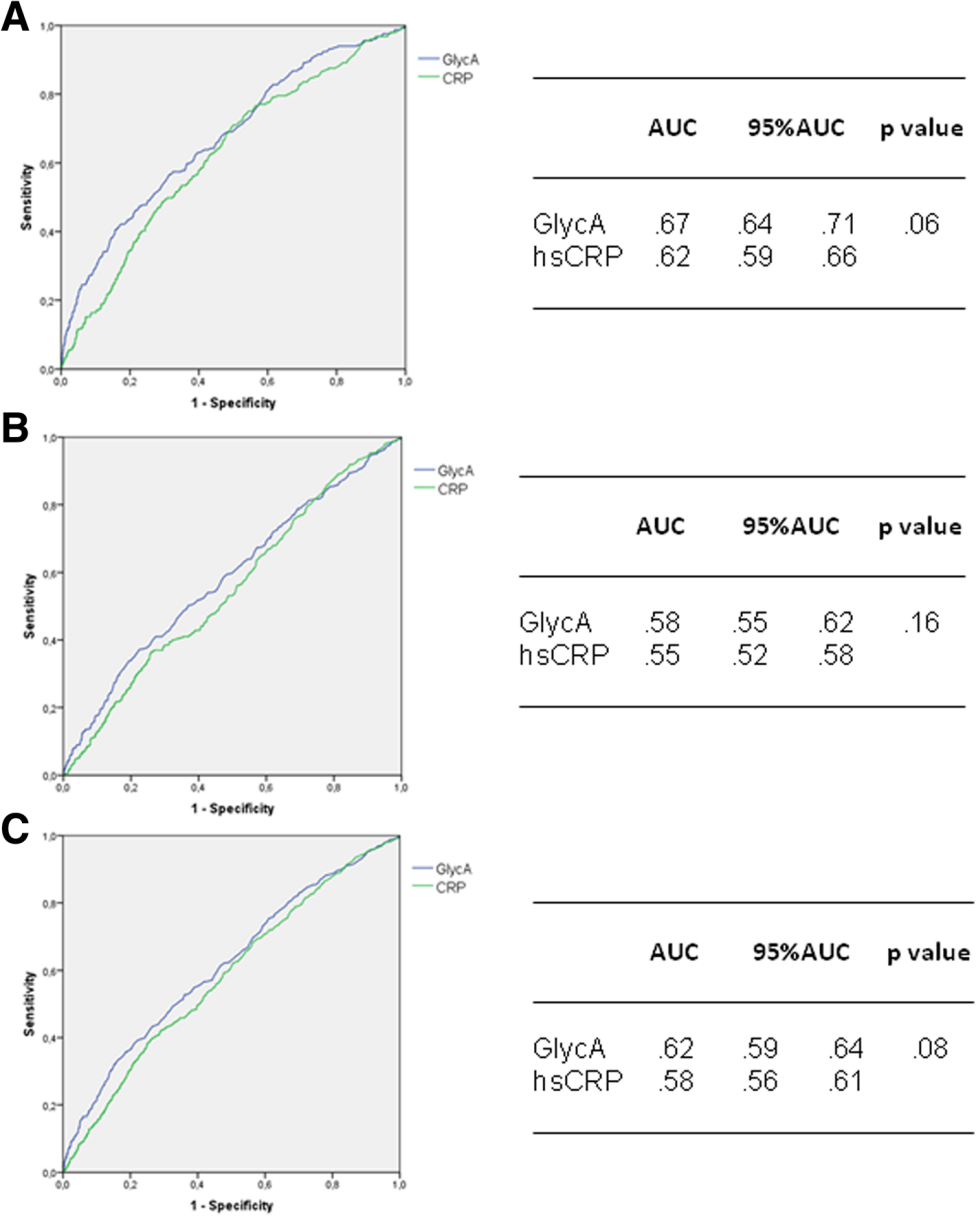
ROC curves on the diagnosis of albuminuria (**a**), eGFR <60 ml/min/1.73m^2^ (**b**) and both (**c**) for GlycA and hs-CRP among 5050 participants (*p* value for C statistics)

Lastly, Fig. 2 depicts the ROC curves for the diagnosis of albuminuria (albuminuria A2 or A3), for the diagnosis of eGFR < 60 ml/min/1.73m^2^ and for both. GlycA showed a nearly significant higher area under curve in comparison to hsCRP for albuminuria and for the combined diagnosis of albuminuria eGFR < 60 ml/min/1.73m^2^ (c statistics *p* value of 0.06 and 0.08, respectively), but not for eGFR < 60 ml/min/1.73m^2^ alone (c statistics p value of 0.16).

## Discussion

The results of the present study demonstrate that GlycA was significantly and independently related to albuminuria and eGFR in a population of middle-aged men and women. Importantly, this relationship was independent of hsCRP, suggesting that measuring glycosylation could provide additional information to the most widely used inflammatory biomarker. These results are in accordance with previous findings in the literature, which showed that GlycA is related to inflammatory diseases, insulin resistance and DM incidence, cardiovascular disease, and mortality.

The current results have two major implications. First, it hints that GlycA might contribute to CKD assessment and for identification of those at higher risk of CKD progression. Prospective studies are necessary to confirm this hypothesis by evaluating the performance of GlycA to predict renal outcomes. These studies should also evaluate if this new biomarker improves prediction of events in comparison to models using the current established markers of CKD progression.

Secondly, it raises questions on the role of glycosylation on CKD. Protein glycosylation refers to the enzyme-mediated posttranslational process of attachment of glycan chains. It is said to be an N-linkage when the glycan chain is attached to the nitrogen of an asparagine residue or to be O-linkage when the glycan is attached to the oxygen of a serine or threonine residue. While O-linkage glycosylation is more prominent intracellularly and is related to protein signaling and trafficking within the cell, N-linkage is the predominant pattern of glycosylation that occurs in circulating plasma proteins, through the action of glycosyltransferases, glycosidases, and syaliltransferases [25]. Glycosylation patterns are wide, depending on the substrate, enzymes, and monosaccharides available and number of branches being added. Glycosylation is known to alter protein function and is involved in several biological functions such as protein trafficking, protein signaling, ligand-receptor recognition, immunity and distinction between self and non-self and inflammation. For example, glycosylation seems to be important in enhancing adhesion molecules signaling in endothelial cells and marginalization and infiltration of leukocytes through the capillary wall [26]. Glycosylation is a determinant of immunoglobulin function and activation of the complement system [27–30]. Altering glycosylation might be a pathway cancer cells use to escape immunity and avoid apoptosis [31, 32].

While the determinants of glycosylation are not fully understood, research on the area is expanding, with a growing interest in glycomics. However, particularly in Nephrology, studies on glycosylation are incipient. Specific abnormal glycosylation patterns are being investigated in auto-immune and inflammatory diseases such as IgA nephropathy [33, 34] and multiple myeloma [35]. In the context of general CKD, data on glycosylation is scarce. One recent study showed that 14 traits of IgG glycosylation were related to renal function in CKD patients and in monozygotic twin pairs discordant for renal function [36]. These analyses were centered in patterns of IgG glycosylation.

As stated before, GlycA reflects increased glycosylation in acute-phase reactant proteins, more specifically of α1-acid glycoprotein, haptoglobin, α1-antitrypsin, α1-antichymotrypsin, and transferrin. Acute-phase reaction is a systemic response to several conditions such as infection, trauma, surgery, immunological and inflammatory diseases. It is mediated by several cytokines, with a particular emphasis on interleukin-6 as a major stimulator. It occurs in acute and chronic inflammation and involves several phenomena such as neuroendocrine, hematopoietic, metabolic changes and hepatic changes [37]. The liver increases the production of several proteins, such as CRP, amyloid-A,α1-acid glycoprotein, haptoglobin, complement fractions, mannose-binding lectin, coagulation factors, among others (positive acute-phase proteins), while decreasing the concentration of proteins such as albumin, transthyretin and thyroxine-binding globulin (negative acute-phase proteins). In addition to the increase in the positive acute-phase protein concentration, it has been shown that the liver also modifies post-translational processing of these proteins, with an increase in the glycosylation, mediated by cytokines and glucocorticoid [38, 39]. Studies suggest that both the change in the concentration of acute-phase proteins, as the pattern of glycosylation of these proteins might influence the inflammatory and immune-modulatory functions attributable to these proteins [40–42], promoting and intensifying the inflammatory response. Whether the relation between GlycA and eGFR and albuminuria is being mediated by the acute-phase response itself or by the increased glycosylation in the acute-phase response is a question that remains to be answered since in the current analysis these two conditions were inseparable.

A better understanding of determinants and patterns of glycosylation, as a more deep comprehension of how and to what extent glycosylation impacts the biological functions of glycoproteins on chronic inflammatory conditions such as CKD is a promising and needed area of research. Particularly of interest, the association between GlycA and albuminuria, the best “dynamic” marker of CKD risk we have so far, suggests that abnormal glycosilation may be an underlying mechanism mediating inflammation in CKD. This hypothesis leads to questions whether glycosylation could play a role not only in mediating the effect of traditional risk factors for CKD, such as diabetes, insulin resistance, and hypertension, but also be involved in inflammation signaling in primary and secondary glomerulonephritis, diseases where immunological insults are pivotal.

Our study has some limitations. First and most importantly, it is a cross-sectional analysis, and the role of GlycA regarding renal hard outcomes remains to be determined. In addition, the population recruited is essentially a non-CKD population. In order to confirm and better understand the role of GlycA in CKD, the present findings need to be confirmed in other studies, with an emphasis in those addressing CKD incidence and progression. Furthermore, we could not perform other techniques of measuring glycosylation, something that would be interesting to explore and adjust in relation to the GlycA signal.

## Conclusion

In conclusion, the results from this cross-sectional study showed that GlycA is significantly and independently associated with albuminuria and eGFR. These findings support further exploration of the role of glycosylation in CKD progression and risk assessment.

## Abbreviations

AUC: Area under curve
BMI: Body-mass index
CKD: Chronic kidney disease
CKD-EPI: Chronic Kidney Disease Epidemiology Collaboration
eGFR: Estimated glomerular filtration rate
HbA1c: Glycated hemoglobin
HDL: High-density lipoprotein
hsCRP: High-sensitivity C reactive protein
LDL: Low-density lipoprotein
NMR: Nuclear mass resonance
ROC: Receiver operating characteristic curve
SBP: Systolic blood pressure

## References

[1] Otvos JD, Shalaurova I, Wolak-Dinsmore J, Connelly MA, Mackey RH, Stein JH, Tracy RP (2015). GlycA: a composite nuclear magnetic resonance biomarker of systemic inflammation. Clin Chem.

[2] Ritchie SC, Würtz P, Nath AP, Abraham G, Havulinna AS, Fearnley LG, Sarin AP, Kangas AJ, Soininen P, Aalto K, Seppälä I, Raitoharju E, Salmi M, Maksimow M, Männistö S, Kähönen M, Juonala M, Ripatti S, Lehtimäki T, Jalkanen S, Perola M, Raitakari O, Salomaa V, Ala-Korpela M, Kettunen J, Inouye M (2015). The biomarker GlycA is associated with chronic inflammation and predicts long-term risk of severe infection. Cell Syst.

[3] Dungan K, Binkley P, Osei K (2015). GlycA is a novel marker of inflammation among non-critically ill hospitalized patients with type 2 diabetes. Inflammation.

[4] Chung CP, Ormseth MJ, Connelly MA, Oeser A, Solus JF, Otvos JD, Raggi P, Stein CM (2016). GlycA, a novel marker of inflammation, is elevated in systemic lupus erythematosus. Lupus.

[5] Ormseth MJ, Chung CP, Oeser AM, Connelly MA, Sokka T, Raggi P, Solus JF, Otvos JD, Stein CM (2015). Utility of a novel inflammatory marker, GlycA, for assessment of rheumatoid arthritis disease activity and coronary atherosclerosis. Arthritis Res Ther.

[6] Bartlett DB, Connelly MA, AbouAssi H, Bateman LA, Tune KN, Huebner JL, Kraus VB, Winegar DA, Otvos JD, Kraus WE, Huffman KM (2016). A novel inflammatory biomarker, GlycA, associates with disease activity in rheumatoid arthritis and cardio-metabolic risk in BMI-matched controls. Arthritis Res Ther.

[7] Duprez DA, Otvos J, Sanchez OA, Mackey RH, Tracy R, Jacobs DR (2016). Comparison of the predictive value of GlycA and other biomarkers of inflammation for Total death, incident cardiovascular events, noncardiovascular and noncancer inflammatory-related events, and Total cancer events. Clin Chem.

[8] Lawler PR, Akinkuolie AO, Chandler PD, Moorthy MV, Vandenburgh MJ, Schaumberg DA, Lee IM, Glynn RJ, Ridker PM, Buring JE, Mora S (2016). Circulating N-linked glycoprotein acetyls and longitudinal mortality risk. Circ Res.

[9] Gruppen EG, Riphagen IJ, Connelly MA, Otvos JD, Bakker SJ, Dullaart RP (2015). GlycA, a pro-inflammatory glycoprotein biomarker, and incident cardiovascular disease: relationship with C-reactive protein and renal function. PLoS One.

[10] Akinkuolie AO, Buring JE, Ridker PM, Mora S (2014). A novel protein glycan biomarker and future cardiovascular disease events. J Am Heart Assoc.

[11] Connelly MA, Gruppen EG, Wolak-Dinsmore J, Matyus SP, Riphagen IJ, Shalaurova I, Bakker SJ, Otvos JD, Dullaart RP (2016). GlycA, a marker of acute phase glycoproteins, and the risk of incident type 2 diabetes mellitus: PREVEND study. Clin Chim Acta.

[12] Dullaart RP, Gruppen EG, Connelly MA, Otvos JD, Lefrandt JD (2015). GlycA, a biomarker of inflammatory glycoproteins, is more closely related to the leptin/adiponectin ratio than to glucose tolerance status. Clin Biochem.

[13] Gruppen EG, Connelly MA, Vart P, Otvos JD, Bakker SJ, Dullaart RP (2016). GlycA, a novel proinflammatory glycoprotein biomarker, and high-sensitivity C-reactive protein are inversely associated with sodium intake after controlling for adiposity: the prevention of renal and vascular end-stage disease study. Am J Clin Nutr.

[14] Amdur RL, Feldman HI, Gupta J, Yang W, Kanetsky P, Shlipak M, Rahman M, Lash JP, Townsend RR, Ojo A, Roy-Chaudhury A, Go AS, Joffe M, He J, Balakrishnan VS, Kimmel PL, Kusek JW (2016). Raj DS; CRIC study investigators. Inflammation and progression of CKD: the CRIC study. Clin J Am Soc Nephrol.

[15] Kubo S, Kitamura A, Imano H, Cui R, Yamagishi K, Umesawa M, Muraki I, Kiyama M, Okada T (2016). Iso H; circulatory risk in communities study investigators. Serum albumin and high-sensitivity C-reactive protein are independent risk factors of chronic kidney disease in middle-aged Japanese individuals: the circulatory risk in communities study. J Atheroscler Thromb.

[16] Lee BT, Ahmed FA, Hamm LL, Teran FJ, Chen CS, Liu Y, Shah K, Rifai N, Batuman V, Simon EE, He J, Chen J (2015). Association of C-reactive protein, tumor necrosis factor-alpha, and interleukin-6 with chronic kidney disease. BMC Nephrol.

[17] Gupta J, Mitra N, Kanetsky PA, Devaney J, Wing MR, Reilly M, Shah VO, Balakrishnan VS, Guzman NJ, Girndt M, Periera BG, Feldman HI, Kusek JW, Joffe MM (2012). Raj DS; CRIC study investigators. Association between albuminuria, kidney function, and inflammatory biomarker profile in CKD in CRIC. Clin J Am Soc Nephrol.

[18] Weiner DE, Tighiouart H, Elsayed EF, Griffith JL, Salem DN, Levey AS, Sarnak MJ (2008). The relationship between nontraditional risk factors and outcomes in individuals with stage 3 to 4 CKD. Am J Kidney Dis.

[19] Schmidt MI, Duncan BB, Mill JG, Lotufo PA, Chor D, Barreto SM, Aquino EM, Passos VM, Matos SM, Molina Mdel C, Carvalho MS, Bensenor IM (2015). Cohort profile: longitudinal study of adult health (ELSA-Brasil). Int J Epidemiol.

[20] Aquino EM, Barreto SM, Bensenor IM, Carvalho MS, Chor D, Duncan BB, Lotufo PA, Mill JG, Molina Mdel C, Mota EL, Passos VM, Schmidt MI, Szklo M (2012). Brazilian longitudinal study of adult health (ELSA-Brasil): objectives and design. Am J Epidemiol.

[21] Bensenor IM, Griep RH, Pinto KA, Faria CP, Felisbino-Mendes M, Caetano EI, Albuquerque Lda S, Schmidt MI (2013). Routines of organization of clinical tests and interviews in the ELSA-Brasil investigation center. Rev Saude Publica.

[22] Fedeli LG, Vidigal PG, Leite CM, Castilhos CD, Pimentel RA, Maniero VC, Mill JG, Lotufo PA, Pereira AC, Bensenor IM (2013). Logistics of collection and transportation of biological samples and the organization of the central laboratory in the ELSA-Brasil. Rev Saude Publica.

[23] Pereira AC, Bensenor IM, Fedeli LM, Castilhos C, Vidigal PG, Maniero V, Leite CM, Pimentel RA, Duncan BB, Mill JG, Lotufo PA (2013). Design and implementation of the ELSA-Brasil biobank: a prospective study in a Brazilian population. Rev Saude Publica.

[24] Levey AS, Stevens LA, Schmid CH, Zhang YL, Castro AF, Feldman HI, Kusek JW, Eggers P, Van Lente F, Greene T, Coresh J (2009). CKD-EPI (chronic kidney disease epidemiology collaboration). A new equation to estimate glomerular filtration rate. Ann Intern Med.

[25] Lyons JJ, Milner JD, Rosenzweig SD (2015). Glycans instructing immunity: the emerging role of altered Glycosylation in clinical immunology. Front Pediatr.

[26] Barthel SR, Gavino JD, Descheny L, Dimitroff CJ (2007). Targeting selectins and selectin ligands in inflammation and cancer. Expert Opin Ther Targets.

[27] Plomp R, Bondt A, de Haan N, Rombouts Y, Wuhrer M (2016). Recent advances in clinical Glycoproteomics of Immunoglobulins (Igs). Mol Cell Proteomics.

[28] Anthony RM, Ravetch JV (2010). A novel role for the IgG Fc glycan: the anti-inflammatory activity of sialylated IgG Fcs. J Clin Immunol.

[29] Quast I, Keller CW, Maurer MA, Giddens JP, Tackenberg B, Wang LX, Münz C, Nimmerjahn F, Dalakas MC, Lünemann JD (2015). Sialylation of IgG Fc domain impairs complement-dependent cytotoxicity. J Clin Invest.

[30] Quast I, Peschke B, Lünemann JD (2016). Regulation of antibody effector functions through IgG Fc N-glycosylation. Cell Mol Life Sci.

[31] Häuselmann I, Borsig L (2014). Altered tumor-cell glycosylation promotes metastasis. Front Oncol.

[32] Saldova R, Wormald MR, Dwek RA, Rudd PM (2008). Glycosylation changes on serum glycoproteins in ovarian cancer may contribute to disease pathogenesis. Dis Markers.

[33] Suzuki H, Fan R, Zhang Z, Brown R, Hall S, Julian BA, Chatham WW, Suzuki Y, Wyatt RJ, Moldoveanu Z, Lee JY, Robinson J, Tomana M, Tomino Y, Mestecky J, Novak J (2009). Aberrantly glycosylated IgA1 in IgA nephropathy patients is recognized by IgG antibodies with restricted heterogeneity. J Clin Invest.

[34] Moura IC, Arcos-Fajardo M, Sadaka C, Leroy V, Benhamou M, Novak J, Vrtovsnik F, Haddad E, Chintalacharuvu KR, Monteiro RC (2004). Glycosylation and size of IgA1 are essential for interaction with mesangial transferrin receptor in IgA nephropathy. J Am Soc Nephrol.

[35] Chen J, Fang M, Zhao YP, Yi CH, Ji J, Cheng C, Wang MM, Gu X, Sun QS, Chen XL, Gao CF (2015). Serum N-Glycans: a new diagnostic biomarker for light chain multiple myeloma. PLoS One.

[36] Barrios C, Zierer J, Gudelj I, Štambuk J, Ugrina I, Rodríguez E, Soler MJ, Pavić T, Šimurina M, Keser T, Pučić-Baković M, Mangino M, Pascual J, Spector TD, Lauc G, Menni C (2016). Glycosylation profile of IgG in moderate kidney dysfunction. J Am Soc Nephrol.

[37] Gabay C, Kushner I (1999). Acute-phase proteins and other systemic responses to inflammation. N Engl J Med.

[38] Van Dijk W, Mackiewicz A (1995). Interleukin-6-type cytokine-induced changes in acute phase protein glycosylation. Ann N Y Acad Sci.

[39] de Graaf TW, Van der Stelt ME, Anbergen MG, van Dijk W (1993). Inflammation-induced expression of sialyl Lewis X-containing glycan structures on alpha 1-acid glycoprotein (orosomucoid) in human sera. J Exp Med.

[40] Zhang S, Shang S, Li W, Qin X, Liu Y (2016). Insights on N-glycosylation of human haptoglobin and its association with cancers. Glycobiology.

[41] McCarthy C, Saldova R, Wormald MR, Rudd PM, McElvaney NG, Reeves EP (2014). The role and importance of glycosylation of acute phase proteins with focus on alpha−1 antitrypsin in acute and chronic inflammatory conditions. J Proteome Res.

[42] Bergin DA, Reeves EP, Meleady P, Henry M, McElvaney OJ, Carroll TP, Condron C, Chotirmall SH, Clynes M, O'Neill SJ, McElvaney NG (2010). α−1 antitrypsin regulates human neutrophil chemotaxis induced by soluble immune complexes and IL-8. J Clin Invest.

